# The British Hospitals Association: Mr. Wade Deacon's Address

**Published:** 1915-11-27

**Authors:** 


### THE BRITISH HOSPITALS ASSOCIATION.

### Mr. Wade Deacon's Address.

The annual meeting of the British Hospitals Association was held at the Westminster Hospital, London, on Thursday, November 18, 1915. The chair was taken by Mr. H. Wade Deacon, Chairman of the Royal Infirmary, Liverpool. The following members were present: Rev. G. B. Cronshaw, Chairman of the Radcliffe Infirmary, Oxford; Mr. W. J. Morton, Mount Vernon Hospital; Mr. C. S. Risbee, Northampton General Hospital; Mr. A. H. Leaney, Birmingham General Hospital; the Rev. D. S. Paterson, Samaritan Free Hospital; Mr. R. J. Bland, Royal London Ophthalmic Hospital; Mr. W. H. Harper, Wolverhampton Hospital; Mr. J. Courtney Buchanan, Metropolitan Hospital; Mr. H. J. Toulmin, St. Albans Hospital; Mr. Howgrave Graham, Hospital for Epilepsy; Mr. R. A. Owthwaite, Hon. Treasurer; and Mr. Conrad W. Thies, Hon. Secretary.

At the outset of the proceedings, on the motion of the Chairman, seconded by the Rev. G. B. Cronshaw, Mr. F. G. Hazell, secretary-superintendent of the Royal Infirmary, Manchester, was elected a member of the Council in the place of Mr. W. G. Carnt, the late secretary-superintendent, who, to the regret of the Council and the whole hospital world, had been compelled to relinquish work on account of ill-health. Mr. J. Courtney Buchanan was appointed a joint honorary secretary to the Association.

### An Interesting Speech.

Mr. Wade Deacon gave expression to the widespread sorrow caused by Mr. Carnt's illness and suffering. He asked the members to leave the appointment of a president for the ensuing year in the hands of the Council to secure that he shall be actively associated with the place where it may be determined to hold the next conference. In regard to the admission of wounded sailors and soldiers to wards of voluntary hospitals, and the question of payment for such cases, Mr. Deacon referred to the circular letter issued by the Council earlier in the year (see The Hospital, March 6, 1915, p. 501) and to the War Office correspondence (see The Hospital, September 4, 1915, p. xvi.), which recommended hospitals which had not already received payment to apply to the local military authorities to enable them to get the matter placed upon a satisfactory financial basis. The Council had considered the question of freedom from duty on spirits used for medicinal purposes in hospitals, and decided to leave the matter in the hands of the British Medical Association and the Pharmaceutical Society. They relied on the promise of the Chancellor of the Exchequer that provision would be made for these charges so far as the voluntary hospitals were concerned. He mentioned that the War Office had refused to recognise badges for hospital employees. Under Lord Derby's scheme such employees should offer themselves for service and, if accepted, each hospital committee should apply to the local tribunals to exempt such of their employees as they considered indispensable. He dealt at length with the important question of the shortage of resident medical officers at voluntary hospitals, and explained the bearing of the circular letter the Council had issued after an interview with Sir Alfred Keogh. It had become the general practice to employ qualified women doctors in increasing numbers so far as they were available. Failing other remedies, arrangements had been made with local general practitioners to carry on the residents' work. Mr. Deacon made some justly complimentary remarks concerning the value of the life-work of Mr. Alexander Hayes, the late secretary of the Metropolitan Convalescent Institution and the Convalescent Homes Association, and of Mr. Howard J. Collins, for many years the much-valued and highly competent house governor of the Birmingham General Hospital. Both these gentlemen during their lifetime had rendered considerable service to the British Hospitals Association, and their memory would be treasured by all who had an intimate acquaintance with their respective fields of labour and the results attending them.

### Provisional Valuations under the Finance Act, 1910.

At the conclusion of the general meeting, Mr. Sydney A. Smith, F.S.I., read a highly technical and valuable paper entitled "Provisional Valuations under the Finance (1909-10) Act, 1910, as they affect the Voluntary Hospitals."

Mr. Smith had evidently taken infinite pains to do his work thoroughly, and he succeeded in the accomplishment of the object he had in view. Mr. Smith proved a very lucid and admirable sphinx to appeal to, and his difficulties will be grasped by the hospital world when we state that his first questioner was the Rev. G. B. Cronshaw, who presented two or three riddles in connection with the position of the Radcliffe Infirmary. These Mr. Smith solved with zest and clearness, the most important one being probably the question of the claim for an allowance for capital expenditure for improvements and the number of years prior to 1909 during which such expenditure would be taken into account in the Government valuation. This, Mr. Smith advised, would entirely depend upon the character of the improvements. Mr. W. J. Morton, secretary of the Mount Vernon Hospital, Northwood, Middlesex, pointed out that in view of the litigation that had taken place on the point in regard to his hospital, it would appear that nothing could be done until a decision had been given in the High Court on a test case (the Lumsden) now under consideration. Mr. Smith mentioned there were several cases in the Courts relating to valuations, of which the Lumsden was the most important. An Amending Act had been promised, and meanwhile the Government was pressing for payment of duty in a number of cases. In closing the discussion and moving a vote of thanks to Mr. Smith, the Chairman stated that the Royal Infirmary, Liverpool, had obtained the advice of professional valuers in the matter, and he had no doubt other hospitals had, or would, adopt the same course.

Mr. Conrad W. Thies seconded a vote of thanks, which was carried with much cordiality. The meeting concluded with votes of thanks to Mr. Deacon for presiding and to the committee of the Westminster Hospital for the use of their board-room.

